# Intrapancreatic Biliary Ductoplasty for Chronic Pancreatitis-Induced Biliary Stricture: Outcome Analysis of a Novel Surgical Technique

**DOI:** 10.7759/cureus.88095

**Published:** 2025-07-16

**Authors:** M. B. Krishna Prasad Chowdary, Sastha Ahanatha Pillai, Karthikeyan Srinivasan, Villalan Ramasamy, Venkkatesh Sreepathi, Thamarai Kannan Murugesan, Padmanabhan Subbareddiar

**Affiliations:** 1 Department of Surgical Gastroenterology, Madurai Medical College, Madurai, IND

**Keywords:** biliary cirrhosis, biliary stenting, biliary stricture, cholangitis, choledochoplasty, chronic pancreatitis (cp), frey's procedure, intrapancreatic biliary ductoplasty, jaundice, pancreatico-biliary surgery

## Abstract

Background: Biliary obstruction is one of the common complications of chronic pancreatitis (CP). Different operative procedures were described for its management. The present study describes the technique and outcome analysis of intrapancreatic biliary ductoplasty for CP-induced biliary stricture.

Methods: From March 2014 to March 2021, patients who underwent Frey's procedure with biliary ductoplasty (choledochoplasty) for CP-induced biliary stricture were analyzed retrospectively.

Results: During the study period, 148 patients underwent surgery for CP, of which 49 (33%) patients had associated biliary obstruction. Among the 49 patients with biliary obstruction, 43 patients underwent biliary ductoplasty along with Frey’s procedure. On preoperative evaluation, elevated serum bilirubin and alkaline phosphatase were observed in 38 (88%) and 43 (100%) patients, respectively, and biliary stricture in 33 (77%) patients. The median duration of surgery was 250 minutes, with a median blood loss of 300 mL. Postoperative complications developed in five (12%) patients, with a median hospital stay of eight days. With a median follow-up of 29 months, recurrent biliary stricture was noted in one (2%) patient, and three patients had readmission for abdominal pain.

Conclusion: Intrapancreatic biliary ductoplasty is a safe and feasible alternative procedure in patients with CP and biliary obstruction. This procedure is comparable to existing standard procedures without an increase in morbidity or mortality. This procedure seems to have a good long-term outcome with a low risk of recurrent biliary stricture.

## Introduction

Chronic pancreatitis (CP) is characterized by progressive and irreversible damage to the pancreas’s exocrine and endocrine components. CP presents with pain and other complications like pseudocyst, biliary obstruction, or gastric outlet obstruction. Benign biliary obstruction (BBO) secondary to CP may be complicated by biliary stricture. The incidence of BBO in CP accounts for 3% to 46% [1]. However, symptomatic biliary stricture develops in only about 10% of cases [1]. Biliary obstruction in CP could be due to pseudocyst, edema, or periductal fibrosis. After the resolution of an acute episode, pseudocyst and edema usually subside, whereas fixed strictures remain unaltered. Untreated biliary stricture is associated with a risk of cholangitis or biliary cirrhosis.

Management of BBO in CP includes endoscopic biliary duct stenting and operative bypass surgery. Endoscopic stenting may be complicated by stent occlusion, migration, and the need for repeated stent exchanges. About half of the biliary stented patients may ultimately need surgical intervention in the long term. Operative bypass surgery includes choledochojejunostomy, choledochoduodenostomy, Roux-en-Y hepaticojejunostomy, and intrapancreatic biliary ductoplasty.

We report our experience with CP-related biliary stricture managed by intrapancreatic biliary ductoplasty (choledochoplasty). In this technique, after performing head coring of the pancreas, the common bile duct (CBD) is opened so that the bile drains into the cavity of the resected pancreatic head, thereby eliminating the need for separate bilioenteric anastomosis and its related complications. This study aims to analyze the short-term and long-term outcomes of intrapancreatic biliary ductoplasty for CP-induced biliary stricture.

## Materials and methods

The present study is a retrospective analysis of collected data. All patients who underwent Frey’s procedure with intrapancreatic biliary ductoplasty for CP with biliary obstruction from March 2014 to March 2021 were analyzed.

Selection criteria

Inclusion Criteria

Intrapancreatic biliary ductoplasty was performed in patients of CP with a good performance of Eastern Cooperative Oncology Group (ECOG) (0-1) having intractable pain coexisting with either biliary stricture confined to the pancreatic portion of the bile duct or elevated serum bilirubin, or persistently elevated alkaline phosphatase (more than three times the normal levels for more than one month).

Exclusion Criteria

For patients with a previous history of surgery for CP or suspicious/proven pancreatic malignancy or intractable cholangitis or biliary cirrhosis, the proposed surgery was not opted for.

Technical information

**Figure 1:**
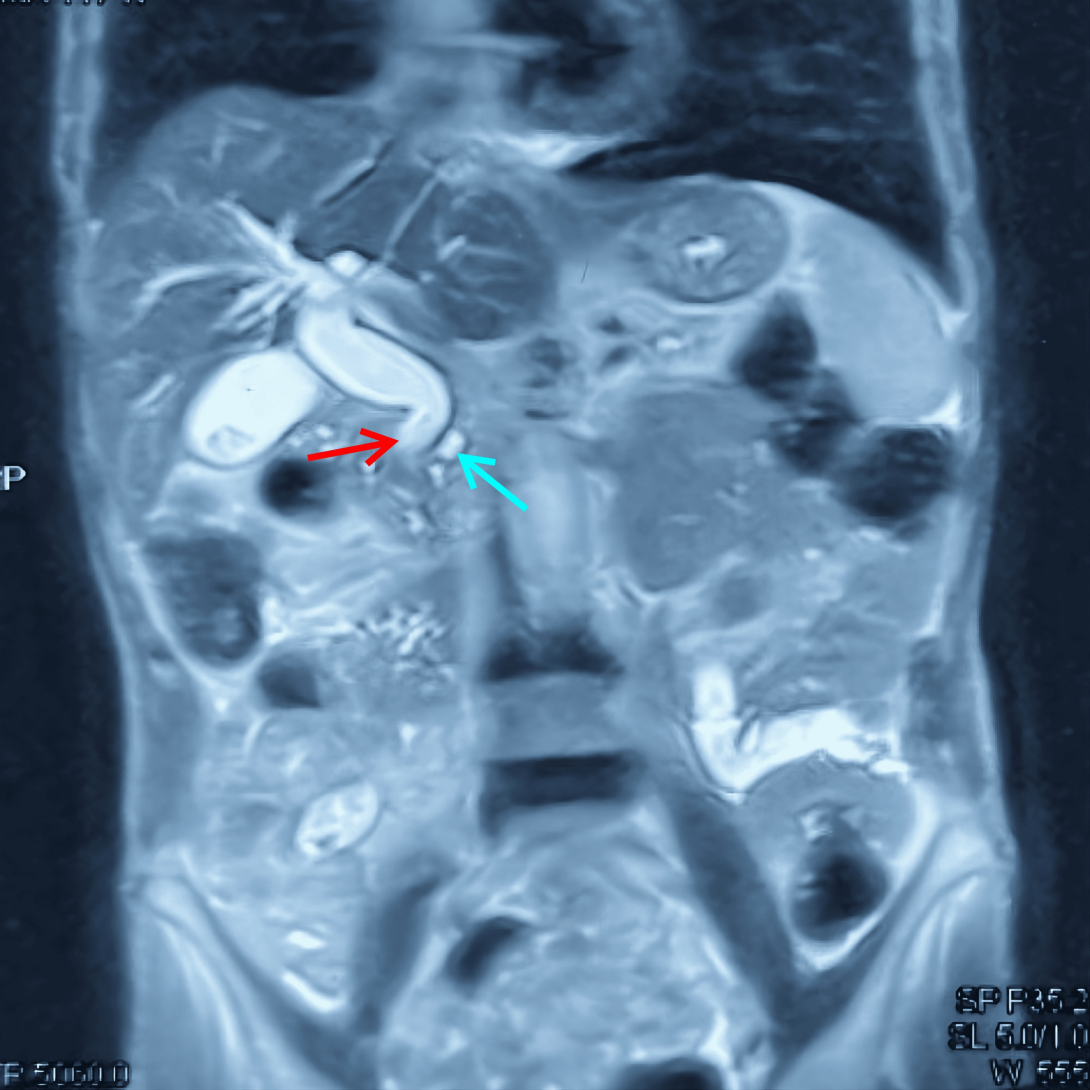
MRI of the abdomen showing biliary stricture secondary to chronic pancreatitis. Red arrow showing biliary narrowing; blue arrow showing dilated pancreatic duct MRI: magnetic resonance imaging

Diagnosis of CP with biliary stricture was made by clinical history and examination, liver function tests (LFTs) (serum bilirubin, serum alkaline phosphatase), contrast-enhanced computed tomography (CECT) of the abdomen, or magnetic resonance imaging/magnetic resonance cholangiopancreatography (MRI/MRCP) (Figure 1). Frey’s procedure with intrapancreatic biliary ductoplasty was performed, and patient outcomes were noted. Analysis was performed with respect to preoperative, intraoperative, postoperative, and long-term outcomes. Patients were followed for the first two years once every three months, thereafter once every six months. Follow-up includes clinical examination, LFTs, and imaging. Patients were followed up on an outpatient basis and by teleconsultation for those who missed the follow-up.

Technique

**Figure 2:**
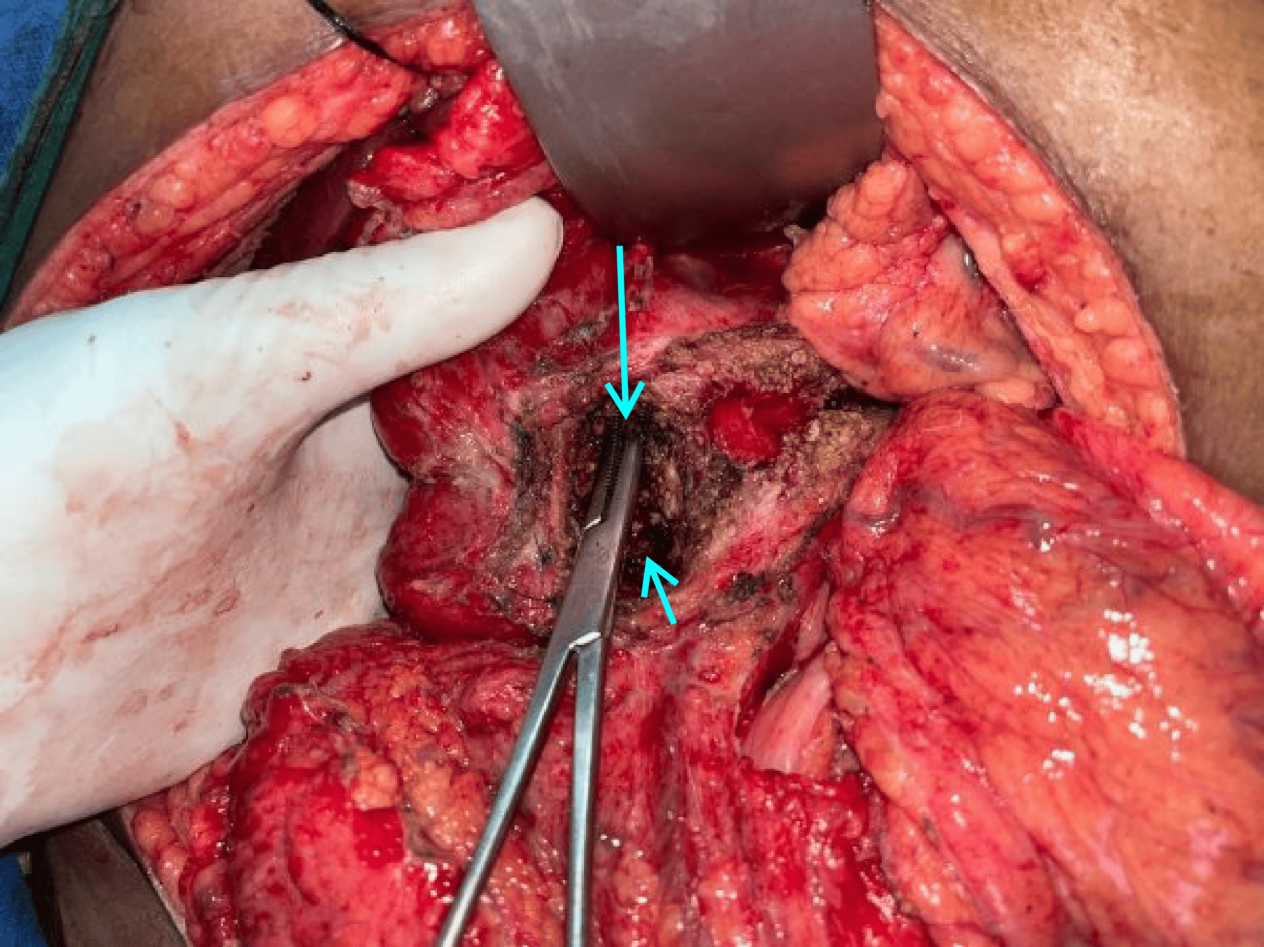
Frey’s procedure with intrapancreatic biliary ductoplasty (artery forceps is seen inside the bile duct). Long arrow depicting the location of the bile duct; short arrow showing the cored out pancreatic head

**Figure 3:**
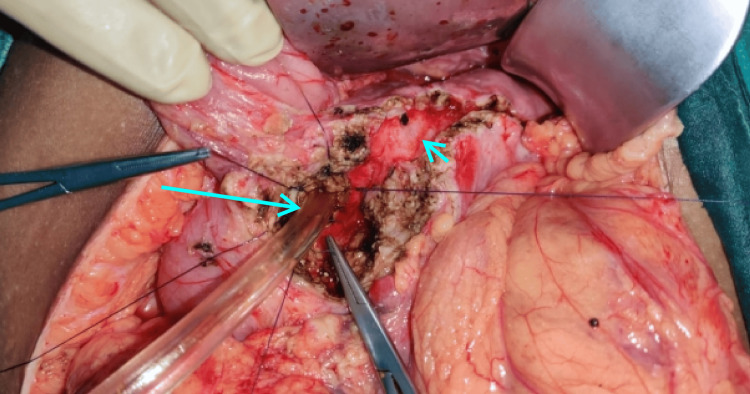
Biliary ductoplasty being performed using 3-0 polyglactin (Vicryl) sutures with a suction cannula inside the bile duct as a guide. Long arrow showing the bile duct guided by the suction cannula; short arrow representing laid opened pancreatic duct

**Figure 4:**
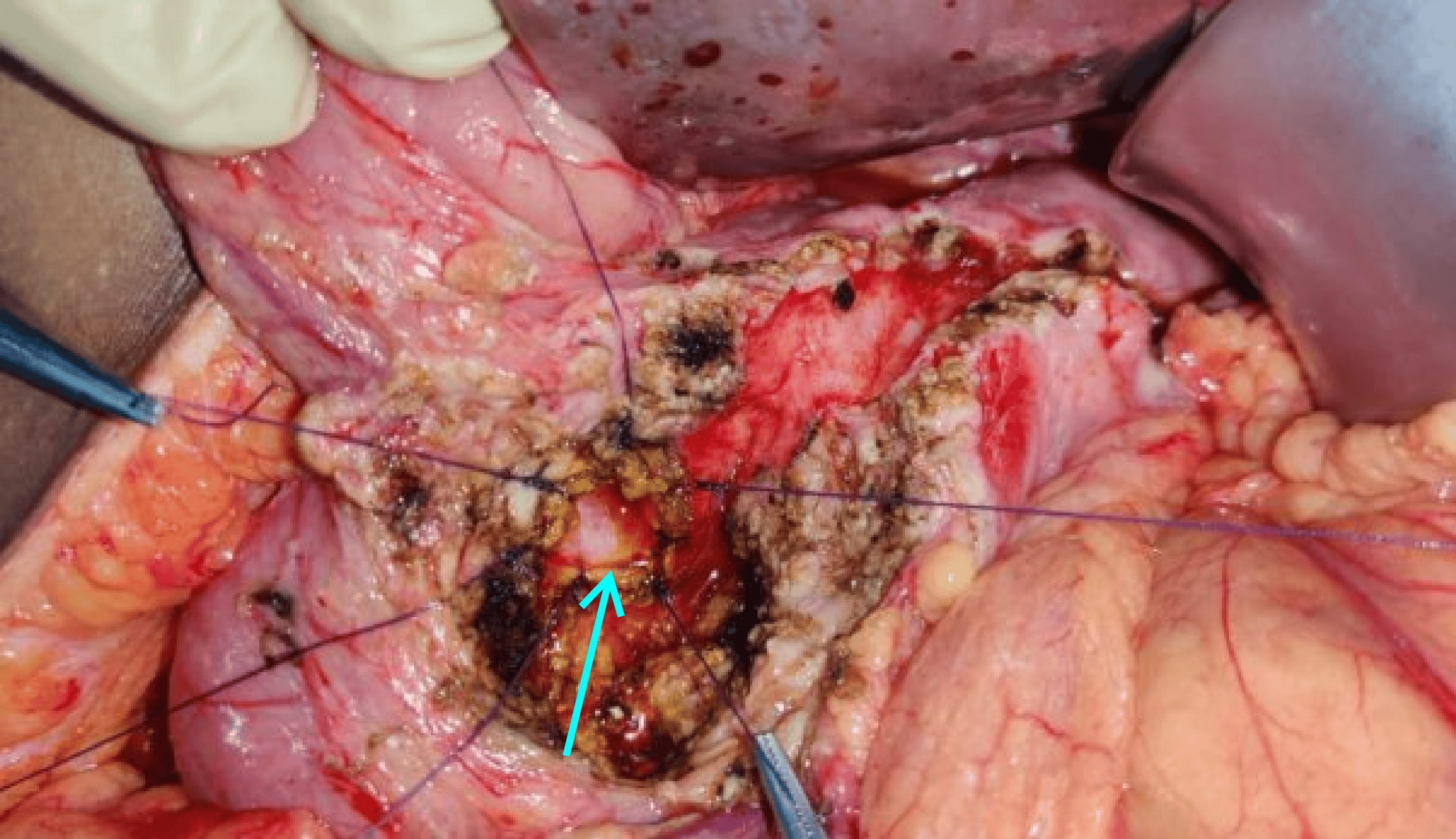
Completed intrapancreatic biliary ductoplasty. Arrow depicting stay sutures at the anastomotic edge

The abdomen was entered through a bilateral subcostal incision, and the pancreas was exposed from head to tail. Head coring was performed in such a way that the thin rim (5 mm) of the pancreas was left along the C loop of the duodenum and to the right of the superior mesenteric vein and portal vein. Posteriorly, coring was performed up to the posterior capsule to expose the CBD. A longitudinal opening was made in the intrapancreatic CBD (Figure 2). The edges of the CBD are sutured superiorly to the rim of pancreatic tissue and inferiorly to the posterior pancreatic capsule using 3-0 polyglactin (Vicryl; Ethicon, Inc., Somerville, NJ, US) sutures in an interrupted fashion (Figures 3, 4). Cholecystectomy was performed in all cases undergoing intrapancreatic biliary ductoplasty. A single-layer Roux-en-Y pancreaticojejunostomy was performed continuously with 3-0 polypropylene sutures with reinforcing interrupted sutures. An end-to-side jejunojejunostomy was performed. Patients were started on an oral diet by the third postoperative day. Drains were removed by day 7 if the drain tube amylase and quality of drains were normal, and patients were discharged on the same day.

Definition

All deaths that occurred within 90 days of surgery or while the patient was still in the hospital were considered postoperative deaths. The International Study Group on Pancreatic Surgery (ISGPS) recommendations were used to describe and categorize pancreatic fistulae, post-pancreatectomy hemorrhage, and delayed gastric emptying [2-4]. Bile leak was defined and classified according to the criteria of the International Study Group of Liver Surgery [5]. Fasting blood sugar levels greater than 126 mg/dL and serum glycosylated hemoglobin A1c (HbA1c) levels greater than 6.5% were used to identify diabetes mellitus (DM). Steatorrhea and/or the requirement to take supplemental pancreatic enzymes were indicators of exocrine pancreatic insufficiency (EPI). Biochemical tests to assess pancreatic exocrine function are not routinely performed.

Statistical analysis

Information recorded on the data collection forms was uploaded to an Excel sheet (Microsoft Corp., Redmond, WA, US), and data were analyzed using IBM SPSS version 20 (IBM Corp., Armonk, NY, US). Continuous variables were presented by mean, SD, median and range, and 25th and 75th percentiles.

## Results

During the study period, 148 patients underwent surgery for CP. Biliary obstruction was found in 49 (33%) patients. Of these, 43 patients were included in the present study. The remaining six patients underwent other forms of biliary drainage and were excluded from the study.

The median age was 51 years (range 33-71 years). Men constituted 31 (72%) and women 12 (28%). The mean body mass index (BMI) was 21.95 ± 2.3. Diabetes was present in 24 (56%) and exocrine insufficiency in 12 (28%). Alcohol intake was noted in 27 (63%) patients (Table 1).

**Table 1:** Demographic and clinical parameters of patients.

Parameters (n = 43)	
Age, median (range)	51 years (33-71 years)
Body mass index, mean ± SD	21.95 ± 2.3
Diabetes mellitus, n (%)	24 (56%)
Exocrine insufficiency, n (%)	12 (28%)
Alcohol intake, n (%)	27 (63%)

Elevated serum bilirubin and alkaline phosphatase were observed in 38 (88%) and 43 (100%) patients, respectively. Imaging revealed biliary stricture in 33 (77%) patients.

The median duration of surgery was 250 minutes (range 200-440 minutes). The median blood loss was 300 mL (range 240-750 mL). Two (5%) patients needed intraoperative blood transfusions (one unit each). Postoperative complications developed in five (12%) patients (Table 2). In the immediate postop period, three (7%) patients developed pancreatic leak, and one of the three patients had associated bile leak. All three patients were managed conservatively. Post-pancreatectomy hemorrhage was seen in two (5%) patients. Both patients were managed with blood transfusions and hemostatic agents without the need for angioembolization or re-exploration. There was no 90-day mortality. The median duration of hospital stay was eight days (range 7-22 days).

**Table 2:** Short-term outcomes.

Short-term outcomes (n = 43)	
Duration of surgery (median)	250 minutes (200-440 minutes)
Blood loss during surgery (median)	300 mL (240-750 mL)
Intraoperative blood transfusion, n (%)	2 (5%)
Postoperative morbidity	5 (12%)
Postoperative pancreatic leak	3 (7%)
Post-pancreatectomy hemorrhage	2 (5%)
Delayed gastric emptying	3 (7%)
Bile leak	1 (2%)
Surgical site infection	3 (7%)
Hospital stay (median)	8 days (7-22 days)
Mortality (90-day)	None

Patients were followed up for a median of 29 months (range 9-60 months). Two patients were lost to follow-up within one year. Three patients developed recurrent abdominal pain requiring hospitalization, and one of them died after three years due to malnutrition. Recurrent biliary stricture was noted in one (2%) patient presenting within one year. None of the patients developed malignancy on long-term follow-up (Table 3).

**Table 3:** Long-term outcomes.

Long-term outcomes (n = 41)	
Median follow-up	29 months
Biliary stricture	1 (2.4%)
Readmission for pain	3 (7%)
Malignancy	None

## Discussion

BBO secondary to CP is one of the common complications with varying incidences reaching up to 46% based on the criteria used for diagnosis [1]. The incidence of biliary stricture in the present study was 33%. The anatomical relationship of the CBD with the head of the pancreas is an important factor influencing the nature of the stenosis in CP. In up to 85% of people, the CBD traverses the pancreatic head, which is the reason for biliary obstruction in CP [6]. The intrapancreatic portion of the CBD varies in length from 1.5 to 6 cm, which accounts for the variability of stricture lengths seen in clinical practice [7]. CBD stricture occurs as a consequence of recurrent acute inflammatory episodes, which may ultimately result in a periductal fibrotic stricture, and this phenomenon is high in the calcific variant [1].

The clinical course of CBD obstruction varies and is characterized by exacerbations and remissions. Pain is the predominant clinical feature in most patients, and jaundice is present in 30%-50% of patients [8]. Jaundice may be transient, recurrent, or persistent [9]. Transient jaundice is typically seen during acute exacerbations, which resolve when the inflammatory process settles. Persistent jaundice is associated with severe fibrosis from cicatrization of the distal CBD and calcification in the head of the pancreas. Raised alkaline phosphatase is the most frequently encountered abnormality in CBD obstruction [10,11]. Cholangitis occurs in 10% of patients [12]. Biliary cirrhosis associated with CBD obstruction was seen in 7.3% in the study by Frey et al. [1], but none had cirrhosis in other similar series [13]. In this study, biliary bypass was performed in five patients without jaundice but with persistently elevated alkaline phosphatase, as these patients may have an increased risk of secondary biliary cirrhosis.

On imaging, these biliary strictures were long with smooth tapering of the distal CBD or stricture at the level of the ampulla with a dilated proximal duct. At times, stricture may extend above into the suprapancreatic CBD due to extensive peripancreatic inflammation. Patients with strictures at the level of the ampulla with a dilated bile duct into the pancreatic parenchyma will be adequately treated by a head-coring procedure with intrapancreatic biliary ductoplasty to ensure bile drainage into the Roux limb. In cases where the suprapancreatic CBD is involved, hepaticojejunostomy is a better surgical option.

Surgical intervention for CP is widely accepted as the most effective therapeutic option for the control of pain and management of complications [14]. Apart from pain, surgical management can mitigate the risk of recurrent acute episodes of pancreatitis or the risk of malignancy [15] and treat biliary obstruction and obstructive gastrointestinal complications [16]. Currently, biliary stenting is at best a temporizing procedure, particularly for cholangitis. As a definitive treatment, biliary stenting may be reserved for patients with serious comorbid disease or those unwilling to undergo surgery. Biliary stenting is associated with problems like stent malfunction with clogging, dislodgement, and septic complications [17-20].

We perform Frey’s procedure for the management of pain in CP as it is associated with good pain relief in 75%-90% of patients with low morbidity and mortality [21-23]. Surgical approaches to treat biliary obstruction due to CP are multiple, with the most performed procedure being hepaticojejunostomy. Hepaticojejunostomy is preferred because of its low failure rate [10,24,25]. Choledochoduodenostomy is not preferred by many as there is a risk of duodenal scarring from recurrent episodes of pancreatitis involving the head of the pancreas. Another procedure for biliary obstruction is intrapancreatic biliary ductoplasty, which was described by Beger et al. in 1989. In the series published by Beger et al., this procedure was performed in 13 patients [26]. A similar technique of opening the bile duct was described by Gloor et al. [27] as a modified technique of the Beger et al. and Frey’s procedure. This procedure was not popular because many thought that a mixed fistula associated with this procedure may increase morbidity. Contrary to the belief, in this study, only three (7%) patients developed a pancreatic leak, of which one patient also had a bile leak. Various studies have shown that Frey’s procedure is associated with a leak rate of around 9%-10% [28,29]. The leak rates in our study were not different from those of other studies analyzing the outcomes of Frey’s procedure. All three patients with leaks settled with conservative management. They required only a delayed removal of the drain and a prolonged hospital stay. The other concern was that an anastomosis performed at the head of the pancreas may be affected by the recurrence of pancreatic inflammation. This may lead to recurrent biliary stricture and obstructive jaundice in the long run. Only two patients were lost to follow-up. The rest 41 patients had a fairly long median follow-up of 29 months. On follow-up, they were evaluated periodically with LFT and imaging. During this follow-up, three patients had admission for pancreatitis, and one patient died at three years due to malnutrition. One patient developed recurrent biliary stricture 11 months after surgery. The operative duration, blood loss, and transfusion requirement are comparable with Frey’s procedure performed in other series [28-30].

We propose intrapancreatic biliary ductoplasty as an alternative procedure for hepaticojejunostomy when a patient is being operated on for pain along with biliary obstruction. Advantages of the current procedure include avoidance of separate bilioenteric anastomosis and the need for dissection of suprapancreatic CBD, which at times may be difficult due to the presence of peri-choledochal collaterals and inflammation. In the event of recurrent biliary stricture after ductoplasty, hepaticojejunostomy may still be feasible.

Limitations

The main limitation of the current study is the lack of literature related to the procedure for detailed comparison. There is a need for a larger sample size to draw in-depth conclusions. In addition, a longer duration of follow-up is required to accurately assess the efficacy of the procedure. A tertiary care setup and an experienced pancreatic surgeon are also required because of the technical complexity and multidisciplinary approach.

## Conclusions

Intrapancreatic biliary ductoplasty is a novel, safe, and feasible alternative procedure in patients with CP and biliary obstruction. It eliminates the need for bilioenteric anastomosis, thereby reducing further complications related to the procedure. There is an added advantage in decreasing bleeding complications in portal hypertension patients. This technique is comparable to existing standard procedures without an increase in morbidity or mortality. This procedure seems to have a good long-term outcome with a low risk of recurrent biliary stricture.
